# MRSA Spinal Epidural Abscess as a Neurosurgical and Infectious Disease Emergency with Unresolved Antimicrobial Solution

**DOI:** 10.1155/2019/7413089

**Published:** 2019-01-30

**Authors:** James Ebot, W. D. Freeman, Robert Wharen, Mark Anthony Diaz, Claudia Libertin

**Affiliations:** ^1^Department of Neurosurgery, Mayo Clinic, Jacksonville, Florida, USA; ^2^Department of Infectious Disease, Mayo Clinic, Jacksonville, Florida, USA

## Abstract

Spinal epidural abscess caused by MRSA, a life-threatening organism resistant to methicillin and other antibiotics, is a rare but important infectious pathology due to its potential damage to the spinal cord. We present the case of a 74-year-old man who hematogenously seeded his entire epidural spinal canal from C1 to sacrum with MRSA bacteria and remained infected even after maximal treatment with vancomycin and daptomycin. Ceftaroline, a new 5th generation antibiotic with recently described clearance of widespread MRSA infection in epidural complex spine infections, was added to vancomycin as dual therapy for his MRSA infection. A 74-year-old diabetic man with prior right total knee arthroplasty and MRSA infection presented with persistent bacteremia and sepsis. He was transferred to our academic center after diagnosis of entire spine epidural abscesses from C1 to sacral levels with midthoracic MRI T2 hyperintensities of the vertebral bodies and disc concerning for osteomyelitis and discitis. Despite surgery and IV vancomycin with MIC of 1, suggesting extreme susceptibility, the patient's blood cultures remained persistently bacteremic at day 5 of treatment. After 48 hours of dual antibiotic therapy with vancomycin and ceftaroline, his blood cultures came back showing no growth. The patient's outcome was unfavorable due to the advanced nature of his infection and multiple comorbidities, but his negative blood cultures after the addition of ceftaroline to his regime require further investigation into this dual therapy. Randomized controlled trials of 5th generation or combinatorial antibiotics should be considered for this disease.

## 1. Introduction

Spinal epidural abscess is a rare but important infectious pathology, and prompt diagnosis and management is paramount to avoid potential complications to the spinal cord such as paralysis. The source of infection may be via hematogenous spread, direct extension from nearby tissues, or iatrogenic, from spinal procedures. The most common pathogen is *Staphylococcus aureus*, identified in about 75% of the cases [1]. Spinal decompression of the epidural abscess coupled with antimicrobial therapy, especially for those with neurologic deficits is the management of choice, but most institutions adopt a multidisciplinary approach to care with treatments tailored to individual patient presentation. The last decade has seen a rise in the incidence of spinal epidural abscesses mostly attributed to increase in spinal procedures and instrumentation, as well as increased use of injected drugs of abuse. Penetration of antistaphylococcal antimicrobials into the epidural space after surgical debridement is imperative for excellent outcomes. Vancomycin penetrates fairly well into inflamed meninges [2]. As vancomycin minimum inhibitory concentrations (MIC) creep higher, careful antimicrobial management is as critical as early and complete debridement of the abscess.

We present a case of a 74-year-old gentleman who was diagnosed with early prosthetic joint infection (PJI) following a right total knee arthroplasty (TKA) which was associated with methicillin-resistant *Staphylococcus aureus* (MRSA) bacteremia. The MRSA bacteremia seeded other joints and the spinal epidural space. Despite prolonged antimicrobial therapy on two occasions and surgical debridement of joints, he developed a large spinal epidural abscess and refractory MRSA bacteremia [3] that required combination of ceftaroline and vancomycin for bacteremia clearance. This case highlights several management features regarding spinal epidural abscesses and the need for awareness of drug penetration into the CNS by providers.

## 2. Case Report

The patient is a 74-year-old male with comorbidities of coronary artery disease, congestive heart failure, hypertension, and renal insufficiency who presented to an outside hospital with back pain and left upper extremity weakness associated with high fevers and urinary incontinence. Outside spinal imaging showed a large spinal abscess; therefore, he was transferred to our institution for a higher level of care. On presentation, the patient was in septic shock requiring fluid boluses and inotropic agents to stabilize him. Further history was obtained from the family as follows:

- On November 2016, he underwent an elective right TKA complicated by an early MRSA PJI associated with bacteremia
- On May 0f 2017, he underwent resection of the hardware in the knee, incision and drainage, followed by 12 weeks of daptomycin therapy
- Five months later, on October of 2017, due to relapse, he had a second debridement of the right knee for source control as well as left ankle incision and debridement followed by another 8 weeks of daptomycin for this relapse of infection
- A daptomycin-susceptible, vancomycin-susceptible MRSA was isolated from blood and both surgical sites, knee and ankle, on both occasions
- He had ongoing thoracic back pain since 2016 which was monitored radiographically by his local providers, until the development of spinal epidural abscess with upper extremity weakness, which prompted his current hospitalization in January of 2018

**Figure 1:**
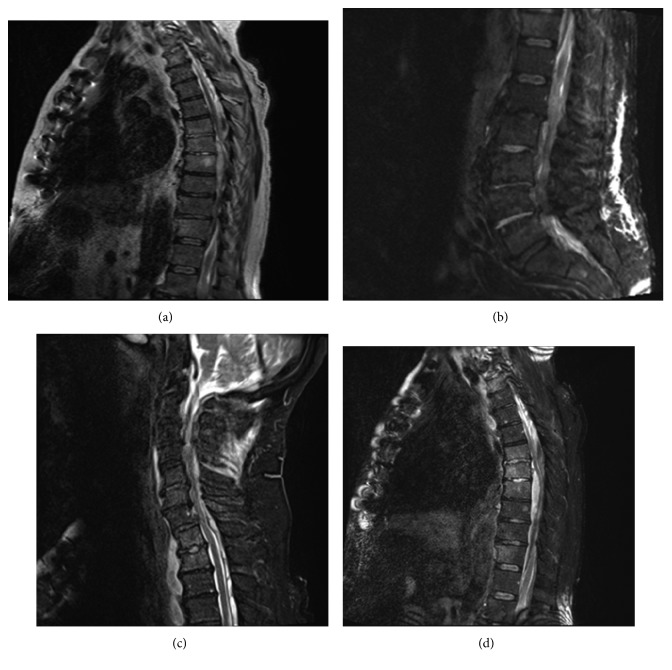
There is extensive epidural phlegmon throughout the cervical (c), thoracic (a, d), and lumbar spine (b) with intracranial extension into the posterior fossa beneath the cerebellum (c). There are small pockets of possible early abscess organization within this phlegmon, but no drainable collection is yet present. The phlegmon causes severe cord compression at C7–T1 (c) and T5–T10 (a, d) levels. There is extension inferiorly through the sacral level (b). There is relative increased T2 signal in the anterior and posterior bony elements at the T1-T2 and T6–T8 levels (d).

Review of systems on presenting to our institution was significant for general weakness and malaise, right shoulder and thoracic back pain, and constipation from narcotics. He was hemodynamically unstable requiring inotropic support. He was awake and oriented, following commands with intact speech. There were no cranial nerve deficits. On motor testing, he had normal muscle bulk with generalized hypotonia. There was no movement of his left upper extremity. He had 2/5 strength on his right upper extremity and 2/5 strength on his bilateral lower extremity. There was decreased sensation to light touch on his left side. Reflexes were globally decreased with negative Hoffman and Babinski signs. The white blood cell count was 30,000/L, and procalcitonin was 4.88 ng/ml. Blood cultures grew MRSA rapidly. Repeat imaging of the brain and spine at our institution showed extensive epidural phlegmon throughout the cervical, thoracic, and lumbar spine with intracranial expansion into the posterior fossa beneath the cerebellum with pockets of possible early organizing abscess within the phlegmon (Figure 1). Brain imaging identified no discrete abscess or leptomeningeal enhancement.

Neurosurgery immediately evaluated the patient and promptly performed a cervical spine decompression of C1–C7 and thoracic spine decompression of T5–T7. Operatively, a large epidural abscess was found, drained, and washed out. He was started on vancomycin every 12 hours with trough vancomycin levels being therapeutic. The patient subsequently underwent irrigation and debridement of the right knee, left ankle, and left great toe as well at our institution; all surgical sites grew MRSA with vancomycin MIC of 1 mcg/ml. Despite attempts at source control and optimal pharmacokinetic dosing of vancomycin with a trough level of 20.5 mcg/ml on day 5, he had refractory MRSA bacteremia. Infectious disease deemed he had failed daptomycin therapy; therefore, ceftaroline 600 mg every 8 hours (MIC of 0.38 mcg/ml) was added to vancomycin. Repeat blood cultures showed clearance of bacteremia after 48 hours of initiation of the combination therapy. His left ankle and right knee continued to yield MRSA. Due to his multiple comorbidities and need for more aggressive source control of his infection, i.e., amputation of the leg, palliative care was sought by the family, and he died a few days later.

## 3. Discussion

Prompt and definitive management by all providers is paramount for successful outcomes and prevention of neurological deficits among those with epidural infections [1]. The above-discussed case of disseminated *S. aureus* PJI involved other joints (ankle and toe) and the epidural space through seeding from the blood stream infection. An early PJI was the original source of the bacteremia which was appropriately managed [4] with resection and prolonged targeted antimicrobial with 12 weeks of daptomycin. However, a relapse occurred after the initial completion of 12 weeks of daptomycin leading to repeat knee debridement, with new incision and drainage of the left ankle followed by an additional 8 weeks of daptomycin. The MRSA isolate was susceptible to daptomycin and never developed resistance to daptomycin. It is difficult to precisely determine when the seeding of the epidural space may have occurred, but the patient complained of new onset of back pain months before presentation to our institution. Back pain is a common symptom among those presenting with epidural abscesses and should be quickly investigated especially in settings where bacteremia precedes the pain. We suspect the epidural abscess occurred weeks, if not months, before his presentation to our institution based on the maturity of the abscess on gross operative observation and the extensiveness of the abscess noted on the MRI (Figure 1). This case highlights that a high degree of clinical suspicion is mandatory to diagnose epidural infections early to avoid poor outcomes and that antimicrobial therapy alone may not be successful in curing MRSA once seeded to the CNS site.

Central nervous system (CNS) infections caused by pathogens with a reduced sensitivity to drugs are a therapeutic challenge. This is particularly true for infections caused by penicillin-resistant pneumococci, methicillin-resistant staphylococci, multiresistant Gram-negative aerobic bacilli, or other organisms that affect primarily the CNS. This patient received daptomycin therapy following each knee surgical debridement. However, data on daptomycin penetration into the CNS is limited [5] as well as its use in CNS infections [6]. Also, providers must have an awareness of the peculiarities of the pharmacokinetics of anti-infectives within the CNS [7]. The intracranial-intraspinal space consists of several compartments. Even in individual regions of one compartment, e.g., cerebrospinal fluid (CSF), strong differences in drug concentrations can occur between the ventricular, cisternal, and lumbar parts of the compartment [8]. The complexities of CNS pharmacokinetics of antibiotics and lack of clinical trials make selection of anti-MRSA antibiotics a dilemma for even infectious disease specialists in such cases. The combination of ceftaroline and vancomycin was used to quickly eradicate the refractory MRSA bacteremia to avoid further dissemination of the pathogen. At our institution, vancomycin was chosen over daptomycin due to its documented penetration into the CNS [9] and then combined with ceftaroline due to reports of synergistic activity between vancomycin and ceftaroline [10]. Rapid bacteremia clearance occurred within 48 hours of the use of both agents. Whether or not it would have been successful in curing the epidural infection remains unknown. For patients with inflamed meninges, bactericidal CSF concentrations of vancomycin against susceptible pathogens are reached during high intravenous doses [2] which was achieved. Ceftaroline, a fifth-generation cephalosporin, had been used as salvage therapy in a case of MRSA epidural abscess [11–15]. Infectious disease felt that ceftaroline may enter the CNS like other cephalosporins, but data to support that hypothesis were lacking. Unfortunately, the patient's family opted for hospice care before further monitoring of the spinal infection could be done.

This case of early MRSA PJI of a TKA with dissemination to other joints and the spine demonstrates the need for early source control of the infection. Without aggressive debridement of the source(s) of infection, which may be at more than one location, relapses occur. This case also unfortunately highlights the importance of early recognition of epidural abscesses to avoid neurologic deficits and the extent to which the abscess can grow (Figure 1). Lastly, infectious disease consultation is indicated and the standard of care for any *S. aureus* bacteremia [16] in that 30 and 90 day mortalities are significantly reduced and management of *S. aureus* bacteremia significantly are improved. We confirmed that the combination of ceftaroline and vancomycin rapidly cleared MRSA bacteremia refractory to vancomycin monotherapy. Regarding CNS penetration of ceftaroline, further investigation is needed to determine its role in the management of MRSA epidural infections.
